# Supplementary material for: MetaRibo-Seq measures translation in microbiomes
Source: Nat Commun. 2020 Jun 29;11:3268. doi: 10.1038/s41467-020-17081-z (PMC7324362; doi:10.1038/s41467-020-17081-z)
Supplement: Supplementary file 10 — Supplementary Data 7 [file 41467_2020_17081_MOESM10_ESM.zip › File2/Confidence_High_Taxonomy/276467_out.krona.html]

Javascript must be enabled to view this page.

members
magnitude
magnitudeUnassigned
count
unassigned
taxon
rank

276467\_out

9

9
2
superkingdom

976
phylum
9

9
class
200643

171549
order
9

family
815
9

816
genus
9


SRS1041147\_contig\_number\_311
2292941
species
1

821

SRS056273\_contig\_number\_contig-100\_0.0
species
1

1
species

SRS013687\_contig\_number\_contig-100\_1.1
47678

species

SRS012969\_contig\_number\_14SRS017821\_contig\_number\_5SRS023526\_contig\_number\_6
2292944
3


SRS147088\_contig\_number\_1656
species
204516
1

species

SRS075078\_contig\_number\_contig-100\_0.0SRS098717\_contig\_number\_96
818
2
